# The timing of antenatal glucocorticoids determines the receptor sensitivity in preterm infants

**DOI:** 10.1210/clinem/dgag066

**Published:** 2026-02-15

**Authors:** Ingmar Fortmann, Jan Hendric Britsemmer, Marianne Lehmann, Natalie Taege, Henrik Oster, Henriette Kirchner, Christoph Haertel, Mariana Astiz

**Affiliations:** Department of Pediatrics, University Hospital Schleswig-Holstein (UKSH), Lübeck 23562, Germany; German Center for Infection Research, Partner Site Hamburg-LüBeck-Borstel-Riems, Braunschweig 23538, Germany; Epigenetics and Metabolism, Institute for Human Genetics, UKSH, Lübeck 23562, Germany; Institute of Neurobiology, University of Lübeck, Lübeck 23562, Germany; Epigenetics and Metabolism, Institute for Human Genetics, UKSH, Lübeck 23562, Germany; Institute of Neurobiology, University of Lübeck, Lübeck 23562, Germany; Epigenetics and Metabolism, Institute for Human Genetics, UKSH, Lübeck 23562, Germany; Department of Pediatrics, University Hospital Schleswig-Holstein (UKSH), Lübeck 23562, Germany; Department of Pediatrics, University of Würzburg, Würzburg 97080, Germany; Institute of Neurobiology, University of Lübeck, Lübeck 23562, Germany; Circadian Physiology Lab, Achucarro Basque Center for Neuroscience, Leioa 48940, Spain; Ikerbasque, Basque Foundation for Science, Bilbao 48940, Spain

**Keywords:** prenatal glucocorticoids, premature infants, glucocorticoid receptor, peripheral blood mononuclear cells

## Abstract

**Context:**

The global preterm birth rate is about 11%. Most premature infants (PI) receive antenatal glucocorticoids (aGCs) to accelerate fetal lung maturation; however, this treatment increases the vulnerability for immune disorders later in life.

**Objective:**

We hypothesized that aGCs given at the “wrong” time of day determines vulnerability. We compared the effect of aGCs in PIs exposed in the morning (in-phase, IP with the maternal circadian rhythms) and in the evening (out-of-phase, OP) to controls (C).

**Methods:**

We collected data and blood samples from infants enrolled in a population-based cohort study. Groups are balanced by gestational age, birth weight, and sex. Infants were divided depending on the time of maternal cortisol peak. Exclusion criteria were established by the cohort; additionally, infants born before gestational week 24 and exposed to postnatal GCs were excluded. The study was run in subcohorts; GC receptor (GR) sensitivity was assessed in 6 IPs and 6 OPs. RNA sequencing, RNA expression, and DNA methylation were assessed in peripheral blood cells from 13 Cs, 17 IPs, and 17 OPs. All samples were obtained on average 10 days after birth.

**Results:**

OP infants exhibited reduced GR sensitivity and suppressed expression of genes involved in antibody production, leukocyte migration, antigen presentation.

**Conclusion:**

aGC exposure aligned with maternal GC rhythms; therefore, it would be advisable to improve PIs' capacity to fight against pathogens during the first weeks of life.

Approximately 15 million babies are born too early each year, corresponding to a global preterm birth rate of about 11% (1). While survival and neurological prognosis improve with advancing gestational age and birth weight, outcomes strongly depend on the early environment/treatments to which they are exposed (2, 3). Antenatal glucocorticoid (aGC) therapy is indicated to more than 90% of mothers at risk of preterm delivery between 24 and 34 weeks of gestation. Synthetic GCs (GCs) accelerate fetal lung maturation and reduce the risk of respiratory distress syndrome, the leading cause of mortality in preterm infants (4). While being beneficial for infants' survival, aGC treatment has been associated with an increased vulnerability to develop neurobehavioral and immune disorders later in life, effects attributed to permanent changes in stress axis activity and in the homeostasis of endogenous GCs (5-10).

GCs are steroid hormones with widespread physiologic actions, modulating stress and metabolic and immune responses on activation of nuclear receptors that mainly act as transcription factors (reviewed in (11)). The GC receptor (GR) is expressed in a wide variety of cells, evoking cell-type–specific transcriptional responses in magnitude and directionality (11). GCs are produced by the hypothalamus-pituitary-adrenal axis with a circadian pattern, peaking before the active phase (ie, morning in humans) (12). During pregnancy, only 10% of the maternal GCs cross the placenta driving essential growth and tissue maturation (13) while also providing circadian timing cues to the fetus (14, 15). We have previously shown that the circadian phase of aGC exposure is relevant for long-term neurodevelopmental outcomes (16). Both in mice and preterm infants, the administration of pharmacological doses of synthetic GCs out of phase (ie, evening in humans and misaligned with the maternal physiological GC rhythms) leads to an impaired regulation of the stress axis (ie, hyperactivity, anxiety, low stress-coping capacity) later in life. However, when the administration was in phase (aligned with the maternal GC rhythms), the offspring showed a behavioral phenotype comparable to nonexposed individuals. In the mouse model, we found that these long-lasting changes are explained at the molecular level by an altered GC homeostasis including reduced sensitivity of the GR (16).

In the present study, we assessed the effect of antenatal administration with GCs in preterm babies considering the time of day of the exposure with special focus on the potential differences in GR sensitivity and its functional consequences. The sensitivity of the GR is regulated by several mechanisms (reviewed by (17-20)), including interaction with a multiprotein complex that anchors the GR in its inactive form in the cytosol. This multiprotein complex, including chaperones (heat shock protein 90 [HSP90], HSP70) and immunophilins (FK506-binding protein 5 [FKBP5] and FKBP4), prevent GR from acting as a transcription factor in the absence of GCs, while maintaining a conformation with high hormone affinity (21, 22). In the presence of GCs, the complex GR-GC translocates to the nucleus, binds to GC-responsive elements, and induces/represses gene expression (23, 24). Interestingly, the expression of *FKBP5* is induced by GR-GC, favoring an intracellular negative feedback loop in which FKBP5 protein inhibits the nuclear translocation of GR, thus, being key in controlling its sensitivity (25-28).

Here we first assessed general transcriptional changes in peripheral blood mononuclear cells (PBMCs) from preterm infants exposed to aGCs and term controls (Cs). We found profound transcriptional changes and, interestingly, a lower inferred activity of the GR in exposed infants. Therefore, we assessed GR sensitivity in preterm infants focusing now on the time of aGC exposure. We found that preterm infants exposed to GCs in the late evening (out-of-phase) exhibited reduced GR sensitivity compared to infants exposed in the morning (in-phase). While the main regulator of GR sensitivity, *FKBP5*, was expressed at a similar level between the groups, we found differential levels of methylation in regulatory regions of the FKBP5 promoter. These regulatory sequences serve as binding sites of transcriptions factors (TFs) upstream of several innate and adaptive immunity pathways. By comparing the transcriptome of PBMCs from babies depending on the time of GC exposure, we found pronounced changes in the expression of genes involved in antibody production and B-cell signaling, leukocyte migration and proliferation, antigen presentation, and cellular stress responses in those exposed out of phase. Our data suggest that aGCs in the morning, in-phase and aligned with maternal GC rhythms, would be advisable in the clinic to minimize the altered expression of those immune pathways and likely improve the capacity of preterm infants to fight against pathogens during the first weeks of life.

## Materials and methods

### Premature babies' cohort

Data and blood samples for the present study were obtained from a population-based multicenter cohort study. The IRoN (ImmunoRegulation of the Newborn) study is a prospective observational cohort designed to characterize immune regulation and adaptation, microbiome development, and infection-related outcomes in term and preterm infants, with the overarching aim of defining individual risk profiles for adverse neonatal outcomes in relation to multilevel exposures (environmental, medical, infectious, and pharmacological, both antenatal and postnatal). Longitudinal collection of blood samples (among others) and comprehensive analyses of immunoregulatory and molecular factors were prospectively planned, including cellular immune profiling, microbiome sequencing, and additional molecular assays related to perinatal exposures. The study protocol explicitly covered the assessment of immunoregulatory mechanisms, modifiable perinatal exposures, and their association with short- and long-term clinical outcomes. Since around 90% of preterm infants are exposed to aGCs, our study focused on this major pharmacological intervention. While GR sensitivity, DNA methylation, and transcriptomic profiling were not specified as primary end points in the original protocol, these analyses were conducted as predefined secondary exploratory investigations to environmental/drug exposure within the approved framework. Accordingly, the readouts reported here constitute secondary exploratory analyses within the approved cohort. The fresh blood samples and isolated PBMCs used for the present analyses exclusively consisted of remnant material from clinically indicated or protocol-defined study samples; no additional blood draws were performed for this project. The blood volume (<1% of body blood volume per blood sampling) was in line with current guidelines of the European Medical Agency on the investigation of medicinal products in term and preterm infants; Committee for Medicinal Products for Human Use and Pediatric Committee (PDCO, 2009). The IRoN study was approved by the local ethics committee (approval No. IRoN AZ 2024_222, before AZ15_304). The informed consent signed by parents enrolling their babies in the IRoN study informs about the possibility that biomaterials and data could be passed on, in anonymous form, to other institutions for research purposes only. For this study, we retrospectively collected data from the IRoN study on gestational age at birth, birth weight, sex, and the timing of antenatal synthetic GC (betamethasone, 8 or 12 mg) injections—at least 2 injections 24 hours apart—given to the mother between weeks 24 and 34 of gestation. Depending on the time of maternal physiological cortisol peak (∼at 0800 hours (12)) and the time of antenatal betamethasone injection, infants were divided into 2 groups: the in-phase group, injected between 0600 hours and 1200 hours, and the out-of-phase group, injected between 1800 hours and 0000 hours. The infants from the C group were not exposed to aGCs because they were born at term. Exclusion criteria were those established by the IRoN cohort and additionally dexamethasone (DEX) treatment (because doses are given every 12 hours), single betamethasone treatments, birth before gestational week 24, and postnatal GCs treatment. This study was run in 3 subcohorts described in Table 1. Briefly, the GR sensitivity assay was performed on n = 6 fresh blood samples per group entirely based on the availability. The RNA and DNA samples were obtained from PBMCs. For RNA sequencing, RNA expression and DNA methylation assays, we included: n = 13 Cs, n = 17 in-phase, and n = 17 out-of-phase infants. The assessment of the immune cell profile was conducted within the IRoN study including n = 28 in-phase and n = 48 out-of-phase infants. All samples included in this study were obtained on average 10 days after birth.

**Table 1:** Subcohort descriptors

Variable	In phase	Out of phase	Controls	*P*
PBMCs for RNA/DNA isolation				
Cohort size	17	17	13	
Birth weight, mean ± SD	1447.94 ± 598.32	1638.76 ± 684.60	3577.5 ± 430.94	***^a^P* < .0001; *^b^P* = .2936**
Gestational age, mean ± SD	30.60 ± 3.11	29.88 ± 3.34	39.19 ± 1.35	***^a^P* < .0001; *^b^P* = .6156**
Sex, % males	23.52	64.7	46.15	
Immune cells assessment				
Cohort size	28	48	—	
Birth weight, mean ± SD	1226 ± 88.87	1179 ± 51.10	—	***^b^P* = .891**
Gestational age, mean ± SD	29.11 ± 0.56	28.89 ± 0.39	—	***^b^P* = .738**
Sex, % males	42.8	43.7	—	
GR sensitivity assay				
Cohort size	6	6	—	
Birth weight, mean ± SD	1103.33 ± 487.10	1176.66 ± 296.89	—	***^b^P* = .484**
Gestational age, mean ± SD	26.83 ± 3.18	29.16 ± 1.47	—	***^b^P* = .1082**
Sex, % males	66.67	33.33	—	

Abbreviations: aGCs, antenatal glucocorticoids; GCs, glucocorticoids; GR, glucocorticoid receptor; PBMCs, peripheral blood mononuclear cells.

^*a*^Control vs aGCs.

^*b*^In-phase vs out-of-phase.

### Glucocorticoid receptor sensitivity

GR sensitivity was assessed by an ex vivo assay in which fresh blood samples were incubated with lipopolysaccharide (LPS, L2630) to activate the production of the proinflammatory cytokine interleukin-6 (IL-6). We used 12 fresh blood samples (remnants of the samples routinely taken for diagnostics depending on availability) from babies exposed to aGCs either in-phase or out-of-phase (6 per group; see Table 1). Babies were born on average 10 days before this sample was taken always between 0800 hours and 1000 hours in EDTA-coated tubes. A 50-μL aliquot was incubated with 100 ng/mL of LPS and with LPS and DEX (D2915, Sigma-Aldrich) at different concentrations (0.01, 0.1, and 1 μM) for 12 hours at 37 °C. After incubation, samples were centrifuged (10 000*g* for 10 min) and IL-6 production quantified in duplicates by enzyme-linked immunosorbent assay in the supernatant (EH2IL6, RRID: AB_3731284, ThermoFisher).

### Peripheral blood mononuclear cell isolation from blood

To isolate PBMCs from whole neonatal blood, we used density-gradient centrifugation with Ficoll. Remnants of EDTA blood samples taken between 0800 hours and 1000 hours, from babies born an average of 10 days before, were used to isolate PBMCs. Blood samples from n = 13 Cs, n = 17 in-phase, and n = 17 out-of-phase infants were diluted 1:1 with phosphate-buffered saline, layered over the density gradient medium, centrifuged at 1000*g* for 20 min at room temperature (RT) with the brake off. PBMCs form a distinct cloudy band at the interface that was carefully removed, transferred in a new tube, washed with phosphate-buffered saline and centrifuged at 400*g* for 10 min at RT. The supernatant was discarded, and the cell pellet used for DNA and RNA isolation.

### DNA and RNA isolation

Genomic DNA and total RNA were simultaneously extracted from babies' PBMCs frozen samples using the AllPrep DNA/RNA/miRNA Universal Kit (80224, Qiagen) following the manufacturer's protocol. Briefly, PBMC samples were thawed on ice and centrifuged for 10 min at 4 °C and 20 000*g*. The pellet was homogenized in RLT Plus sample buffer, and genomic DNA and RNA were isolated according to the manufacturer's instructions. The concentration and purity of samples were measured using Epoch Microplate Spectrophotometer. Total RNA was stored at −80 °C and DNA was stored at −20 °C.

### DNA methylation assay

A total of 500 ng of genomic DNA was converted to bisulfite DNA (bisDNA) using the EpiTect Fast DNA Bisulfite Kit (59802, Qiagen) according to the manufacturer's instructions and eluted in 50 µL of EB buffer (provided in the kit). Subsequently, 10 ng of bisDNA were amplified at 56 °C for 40 cycles with the PyroMark PCR Kit (978801, Qiagen) using the respective forward and reverse primers for each CpG-assay (NR3C1 [GR]: Fwd bisPCR: TTTTTGGGGAGGTTTTAGGG; Rev bisPCR: [biotin]TCTACTTTACAACTTCTCTCCCAATA and FKBP5: Fwd bisPCR: GGTTATTGTGAGGATGATAGTAGTT; Rev bisPCR: [biotin]CCCAAACCTATACTACCTACCAA). Quality of amplification was assessed in 1% agarose gel electrophoresis. Pyrosequencing was performed on the PyroMark Autoprep Q48 (9002471, Qiagen) using the Q48 Advanced CpG Reagents according to the manufacturer’s instructions and the perspective sequencing primer (NR3C1 Pyrosequencing primers: GGGAGGTTTTAGGGAA; FKBP5 Pyrosequencing: TTGGGGGTGGGGATT). Sequencing results that did not pass quality control were discarded (NR3C1 [GR]: 3 Cs, 1 in-phase, and 1 out-of-phase; FKBP5: 4 from each group). Thus, we then obtained data from n = 10 Cs, n = 16 in-phase, and n = 16 out-of-phase infant samples in the NR3C1 (GR) methylation assay. In the case of FKBP5, we obtained methylation assay data for n = 9 Cs, n = 13 in-phase, and n = 13 out-of-phase infant samples.

### RNA sequencing and data analysis

Total RNA samples were sent to Novogene, where messenger RNA (mRNA) quality was checked to confirm suitability for sequencing. RNA libraries were then prepared via poly(A) selection and reverse transcription into complementary DNA, followed by library quality assessment. Sequencing was performed using 150 bp paired-end reads on an Illumina PE150 platform in n = 13 Cs, n = 17 in-phase, and n = 17 out-of-phase infant samples. Quality of sequencing was assessed with fastqc (v0.12.0), and fastq files were mapped to the GRCh38 human reference genome with HISAT2 (29). Subsequently, the gene expression matrix (features × samples) was filtered for not-expressed genes by excluding all genes that are not detected in 5% of all samples (counts = 0 in > 2 samples) followed by an additional filtering for low-expressed genes excluding all genes that display less than an average of 10 counts to confirm reliable and robust quantification. A total of 13 866 genes were ubiquitously expressed across all samples. Differential expression analysis was performed in DESeq2 (v1.46.0). For pair-wise comparison between aGC-exposed and C infants, differentially expressed gene analysis was adjusted for covariates of birth weight and gestational age, since groups displayed statistically significant differences for both confounding factors (Supplementary Fig. S1A and S1B (30)). This was achieved by including both parameters in the generalized linear model design (GLM) of DESeq2 as covariates (design = ∼gestational age + birthweight + aGCs). Birth weight values were missing for 1 C child (C14), and gestational age values were missing for 1 C child (C14) and 3 out-of-phase children (OP5, OP6, OP12). Missing values were mathematically imputed with the mean of each corresponding group as described previously (31). Genes were considered differentially expressed with an abs(log2FC) greater than 1 and *P* value less than .05. Subsequently, raw counts were variance stabilized using variance-stabilizing transformation for principal component analysis. For gene set enrichment analysis (GSEA) to identify gene ontology (GO) terms and transcription factor activity analysis, genes were ranked in descending order based on log2fold change * –log10(*P* value) and GSEA for GO terms was performed with clusterProfiler (v4.14.6). GSEA results were adjusted for multiple testing using the false discovery rate (0.05). The top active and suppressed GO pathways based on the normalized enrichment score from each pair-wise comparison were displayed in dot plots. For collapsing related enriched GO terms within a superior parental term, rrvgo (v1.18.0) was used. In brief, a semantic similarity measure was performed, including relative information content. GO terms with a similarity greater than 0.7 were grouped together to a parental term. Then, original GO terms were plotted in a heat map displaying their normalized enrichment score and their attributed parental term as colored annotations. For the analysis of inferred transcription factor activity, the variance-stabilizing transformation counts were included in an univariate linear model (ulm) calculated with the decoupleR (v2.12.0) package as described previously (32). As a transcription factor network, we used the annotated DoRothEA database for human transcription factor-gene interactions from CollecTRI (33).

### Analyses of lymphocyte subsets and regulatory T cells

Immunological analyses of the preterm infant samples were performed within the first 4 days of life. Samples were taken from n = 28 in-phase and n = 48 out-of-phase infants, always between 0800 hours and 1000 hours and processed as previously described by our group (34, 35). Briefly, EDTA whole-blood samples were stored for a maximum of 24 hours at RT before processing at two different laboratories. First, the central laboratory of the university hospital performed the determination of lymphocyte subsets (CD8^+^/CD19^+^) using a BD FACS Canto II system (BD Bioscience); BD FACS Canto Clinical software using Multi test 6-Color and TBNK (T cells and B cells) kits, as well as Multi test CD3/CD8/CD38/HLA-DR kits according to the manufacturer's instructions. Second, the flow cytometric determination of FoxP3^+^ regulatory T cells (T_regs_) was performed in the research laboratory of the pediatric department (36). T_regs_ were determined by their position in the forward-/side-scatter plot (size/granularity) and coexpression of CD3, CD4, CD25, and FoxP3. Fluorescence minus one Cs were used to establish gating boundaries and to identify any background spread of fluorochromes.

### Transcription factor binding prediction

To assess whether the differences in the methylation levels might have a functional relevance, we used Ciiider (Cluster Identification of cis-regulatory elements). Ciiider is a bioinformatic tool designed to predict potential TF binding sites within a region of interest. It uses position weight matrices derived from curated databases, JASPAR in this case, to scan promoter or intergenic regions associated with the genes of interest. It then evaluates motif representation against a reference set, identifying in this way enriched binding sites (37).

### Statistical analysis

For one-to-one comparison, statistical difference were assessed by 2-tailed *t* test after confirming normality by the Shapiro-Wilk test. If normality was not confirmed, we used the Mann-Whitney test. To assess statistical differences between 3 groups, we used 1-way analysis of variance (ANOVA) followed by the Holm-Sidak multiple comparison test, after confirming normality by the Shapiro-Wilk test. If normality was not confirmed, we used the Kruskal-Wallis test followed by Dunn's multiple comparison test. GR sensitivity was assessed by 2-way ANOVA to assess treatment effect, group effect, and the interaction effect, followed by Sidak's multiple comparison test. *P* values less than .05 were considered statistically significant. For the sequencing results, false discovery rate–adjusted *P* values (*P*adj) less than .05 were considered statistically significant.

## Results

**Figure 1:**
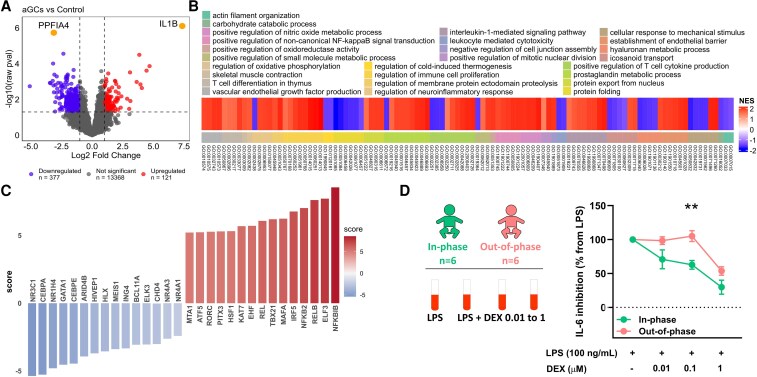
Glucocorticoid (GC) receptor (GR) (NR3C1) activity is reduced by the antenatal exposure to GCs. A, RNA-sequencing experiment was performed from peripheral blood mononuclear cells (PBMCs) obtained from infants antenatally exposed to GCs (aGCs n = 34) in comparison with nonexposed term infants (control n = 13). The differential gene expression is represented in a volcano plot showing that 377 genes were downregulated, and 121 genes were upregulated (*P* < .05; log2fold change < 1) in aGC group compared to controls. B, Gene set enrichment analysis (GSEA) identified the top gene ontology (GO) terms that were activated or suppressed in aGCs infants compared to controls. Genes were ranked descending based on log2fold change*-log10(*P* value) and results were adjusted for multiple testing using false discovery rate (0.05). C, Analysis of inferred transcription factor activity. D, Illustration of sample collection to assess GR sensitivity, fresh blood from n = 6 premature infants per group were incubated with lipopolysaccharide (LPS) in presence of increasing concentrations of dexamethasone (DEX). After 12 hours incubation at 37 °C samples were centrifuged, and interleukin-6 (IL-6) measurements were run in duplicates in the supernatant. The inhibition of LPS-induced IL-6 production is calculated considering LPS treatment as 100%. Data are expressed as mean ± SEM and analyzed by 2-way analysis of variance, treatment effect F(3,40) = 21.15; *P* < .0001; group effect F(3,40) = 0.0001 and interaction effect F(3,40) = 0.0658. Sidak's multiple comparison test showed a statistically significant difference in phase vs out of phase (**P = .0015) for the treatment LPS + DEX 0.1 μM and a tendency (*P* = .0607) for the treatment LPS + DEX 0.01 μM.

With the aim of screening for global effects of aGC administration, we performed bulk RNA sequencing in PBMCs from exposed preterm (aGCs, n = 34) and nonexposed term infants (C, n = 13) and compared the transcriptome. We found that 377 genes were downregulated (*P* < .05; log2fold change < 1), 121 genes were upregulated (*P* < .05, log2fold change > 1), and 13 368 genes did not change significantly in aCG infants compared to nonexposed Cs (Fig. 1A). GSEA for GO terms revealed a statistically significant change in the expression of genes related to general immune and metabolic pathways, as highlighted in the volcano plot (adjusted for multiple testing; *P*adj < .05; Fig. 1B). C infants were born at term and did not receive GCs, therefore, there is a statistically significant difference in birth weight and gestational age between both groups (see Supplementary Fig. S1A-S1C (30)). Interestingly, the differential gene expression in the transcriptome has been calculated after adjusting for birth weight and gestational age, main determinants of the infants’ developing immune system (see Supplementary Fig. S1A and S1B (30)). Furthermore, we found that infants antenatally exposed to GCs showed a lower inferred GR activity (NR3C1) and a high activity for NFKBIB, an inhibitory protein from the nuclear factor κB signaling pathway that is typically repressed by GCs (Fig. 1C). Since we have previously shown in a mouse model that the time of maternal exposure to GCs determined GR sensitivity in offspring (16), we investigated GR sensitivity in the antenatally exposed infants. Estimating the maternal physiological cortisol peak at 0800 hours (12), we assessed samples from preterm infants exposed to GCs in the morning (in-phase group) and in the evening (out-of-phase group). Six fresh blood samples per group were incubated with LPS alone (100 ng/mL) or with LPS in combination with DEX at 0.01, 0.1, and 1 μM for 12 hours at 37 °C (Fig. 1D). The LPS treatment induces the production of the proinflammatory cytokine IL-6. DEX, as an agonist of the GR, suppresses the production of IL-6 in a dose-dependent manner and it is used as a readout for GR sensitivity. As shown in Fig. 1D, samples from preterm infants whose mothers were injected out of phase exhibited reduced GR sensitivity, as higher DEX concentrations were necessary to achieve a comparable inhibition of LPS-induced IL-6 production (see Fig. 1D).

**Figure 2:**
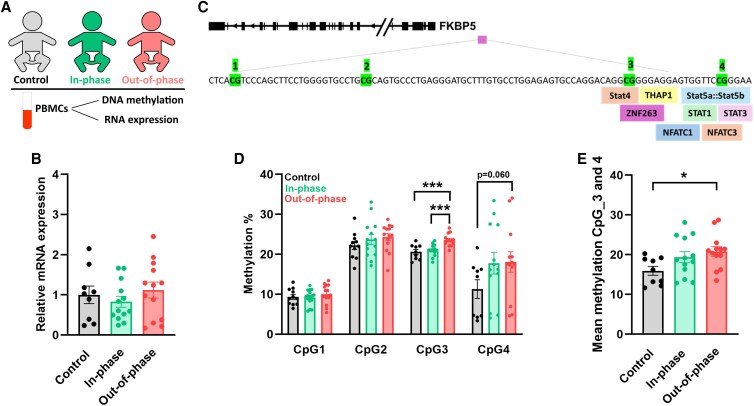
Regulatory regions of *FKBP5* promoter are hypermethylated in preterm infants exposed to antenatal glucocorticoids (aGCs) out of phase. A, Illustration of sample collection, genomic DNA and RNA were isolated from peripheral blood mononuclear cells (PBMCs) obtained from control infants (C n = 13) and preterm infants exposed to aGCs either in-phase (IP n = 17) or out-of-phase (OP n = 17) compared to maternal circadian rhythms. For the comparisons represented here, we considered samples that had both RNA expression values and sequencing data after bisulfite conversion, that is C (n = 9), IP (n = 13), and OP (n = 13). B, Relative *FKBP5* messenger RNA expression was assessed from the RNA-sequencing dataset. Data are expressed as mean ± SEM and analyzed by 1-way analysis of variance (ANOVA) F(2,32) = 0.7144; *P* = .4971. C, Illustration of the regulatory region of FKBP5 where the methylation levels were assessed, the numbers 1 to 4 indicate the position of the 4 CpGs in the sequence. Between CpG 3 and 4, predicted transcription factors binding sites are indicated. D, Percentage of methylation in the CpGs shown in C. Data are expressed as mean ± SEM, CpG3 analyzed by 1-way ANOVA F(2,32) = 11.78; *P* = .0001. Tukey's multiple comparison test: C vs OP; ***P = .0005; and C vs IP; ***P = .0009. CpG4 analyzed by Kruskal-Wallis; *P* = .0592. Dunn's multiple comparison test: C vs OP; *P* = 0.0607. E, Mean methylation level in the CpGs 3 and 4. Data are expressed as mean ± SEM and analyzed by 1-way ANOVA F(2,32) = 3.365; *P* = .0472. Tukey's multiple comparison test: C vs OP; *P = 0.0390.

Since GCs are steroid hormones with widespread physiologic actions and GR is expressed in a wide variety of cells, a reduced sensitivity of GR might have multiple functional consequences. Thus, we aimed to understand how GR differential sensitivity was regulated at the molecular level and the potential functional consequences. In PBMCs, we assessed the mRNA expression levels of *GR* and *FKBP5* (the main regulator of GR sensitivity), as well as methylation levels in key regulatory regions of both genes (Fig. 2A) (38). To this aim, we assessed the relative mRNA expression of *NR3C1* (gene coding for GR) or *FKBP5* between groups and did not find statistically significant effects (Fig. 2B and Supplementary Fig. S2B (30)) in the methylation levels of one of the key regulatory regions of the *NR3C1* promoter (Supplementary Fig. S2C and S2D (30)). Thus, as reported by others (39), the reduced GR sensitivity found in infants exposed to aGCs out of phase cannot be explained by a differential expression of GR. However, when we analyzed the methylation levels in an intronic regulatory region of *FKBP5* (Fig. 2C), we found a significant increase in 2 of the CpGs (3 and 4) in the out-of-phase group (Fig. 2D and 2E). Although the reduced sensitivity of the GR cannot be explained by a differential expression of the activity regulator FKBP5, the higher methylation levels in the regulatory region of *FKBP5* might have functional consequences. Therefore, we used a predictive tool (Ciiider (37), see “Materials and methods”) to identify TF binding sites within this region (see Fig. 2C). Interestingly, we found that CpG3 and 4 were binding sites for TFs upstream several immune, metabolic, and proliferative signaling pathways (STAT1, 3, 4, and 5, THAP1, NFATC 1 and 3, and ZNF263) and that all of them interact with GR signaling in different ways (as reviewed in (40)). We reasoned that the exposure to aGCs out of phase might influence downstream pathways differently and explain an altered sensitivity of the receptor as well as its functional consequences.

**Figure 3:**
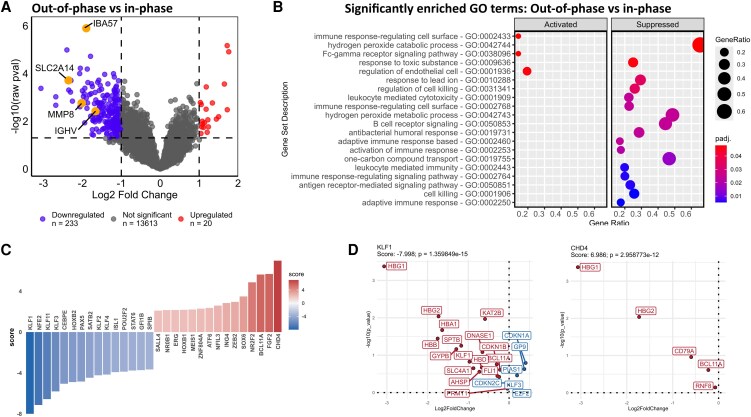
Multiple innate and adaptive immune response pathways are suppressed in preterm infants exposed to antenatal glucocorticoids (aGCs) out of phase. A, RNA-sequencing experiment was performed from peripheral blood mononuclear cells (PBMCs) obtained from infants exposed to aGCs out-of-phase (OP, n = 17)– in comparison with in-phase (IP, n = 17)–exposed infants. The differential gene expression is represented in a volcano plot showing that 233 genes were downregulated, and 20 genes were upregulated (*P* < .05; log2fold change < 1). B, Gene set enrichment analysis (GSEA) identified the top 20 gene ontology (GO) terms that were activated or suppressed in OP compared to IP. Genes were ranked descending based on log2fold change*-log10(*P* value) and results were adjusted for multiple testing using false discovery rate (0.05). C, Analysis of inferred transcription factor activity for the same comparison. D, NR3C1 regulon, depicting genes that are positively or negatively regulated by NR3C1 based on the DoRothEA database are plotted. Most of the genes (red labeled) are aligning with regulation pattern by the univariate linear model.

Next, we used the transcriptome data to compare changes between preterm infants exposed to aGCs out of phase and in phase. We found that 233 genes were downregulated (*P* < .05; log2fold change < 1), 20 genes were upregulated (*P* < .05; log2fold change > 1), and 13 613 genes did not change significantly (Fig. 3A). These two groups did not differ with regard to birth weight and gestational age (Supplementary Fig. S3A and S3B (30)). GSEA for GO terms revealed a significant suppression of more specific immune response pathways such as antibody production and B-cell signaling, leukocyte migration, cytokine production, antigen presentation, and cellular stress responses, as highlighted genes in the volcano plot indicate (see Fig. 3A and 3B). Interestingly, we found that infants antenatally exposed to GCs showed a differential expression of TFs regulating cell proliferation and differentiation depending on the time of day in which they were exposed (Fig. 3C and 3D). Overall, these data suggest that the exposure to aGCs at the “wrong” time of day could reduce GR sensitivity, increase the methylation level on the promoter of one of the main regulators of GR activity (*FKBP5*) where both TFs involved in immune response and GRs interact, leading to an unbalanced reactive state of the immune system.

**Figure 4:**
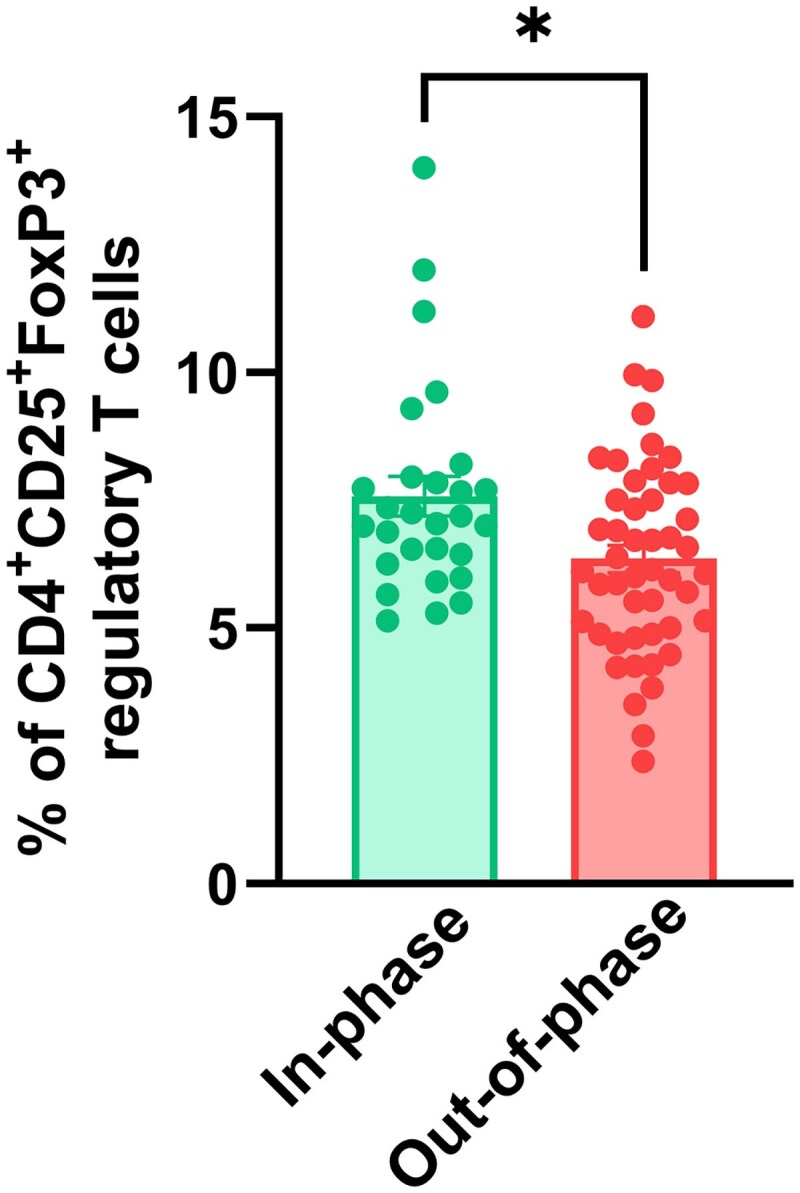
Premature babies exposed to glucocorticoids (GCs) out of phase show lower frequency of regulatory T cells in the first 4 days of life. Frequency of regulatory T cells (CD4^+^CD25^+^FoxP3^+^) in the in-phase (n = 28)– and out-of-phase (n = 48)–exposed groups in the first 4 days of life. Data are expressed as mean ± SEM and analyzed by 2-tailed Mann-Whitney test; *P = .0184.

Our observations at the molecular level align with the lower frequency of T_regs_ (CD4^+^CD25^+^FoxP3^+^) in the out-of-phase group during the first 4 days of life, which could cause a reduced immune tolerance compared to the in-phase–exposed babies (Fig 4). The two groups compared here did not show differences in gestational age, birth weight, sex (see Table 1,) or the frequency of other lymphocytes such as CD3^+^, CD8^+^, CD19^+^, or natural killer CD16^+^/CD56^+^ (Supplementary Fig. S4 (30)).

Finally, our findings suggest that antenatal exposure to synthetic GCs has an inevitable effect on the infant immune system; however, the administration of GCs in the early morning, aligned with the maternal GC rhythms, might have fewer consequences for the preterm infant than in the late evening. Our data provide strong evidence that introducing this timing aspect would be advisable, whenever possible, in the clinic.

## Discussion

The variables that are usually considered to affect the short- and long-term outcomes of aGC treatment in a clinical setting are drug, dose, and regimen, treatment-to-delivery interval, and gestational and individual effects (as reviewed by (41)). Since GCs have a strong circadian rhythm, peaking in anticipation of the active phase, we decided to focus on a less explored aspect: the time of day of the maternal treatment.

In the present study, we first assessed the effect of aGC administration at the transcriptional level in PBMCs from preterm infants and compared them with nonexposed term C infants. Preterm infants have immature immune systems, with reduced innate and adaptive immunity because they are born too early (42). However, the fact that we found significant changes of several immune (eg, *IL1B*, tumor necrosis factor, *CXCL2, CCL3*) and metabolic pathways (eg, *PPFIA4, ACADVL, HAL, ARSA, SLC2A, CKB*) even after adjusting for birth weight and gestational age suggests that aGC exposure could be one of those early factors that further compromise the preterm infants' physiology. The high activity of members of the nuclear factor κB immune signaling pathway, typically repressed by GCs, suggests that a lower GR activity (NR3C1) could result in multiple functional consequences for infants exposed to aGCs. GCs participate in most physiologic processes and the transcriptional responses on GR activation are cell-type specific and can vary in magnitude and directionality (11). Therefore, treatment with aGCs might have an inevitable effect on several aspects of preterm infant development as described by us and others (14, 16). However, the motivation of this study was to explore a way to minimize the negative consequences of this treatment.

Previous evidence from our laboratory showed that indeed premature infants exposed to aGCs out of phase (ie, in the evening) were at a higher risk of developing stress-coping deficits at age 5 years than those exposed in phase (ie, in the morning) (16). Leveraging a mouse model, we explained these long-lasting changes by a basal hyperactivation of the stress axis, impaired negative feedback, low expression of GR in the hypothalamus, and reduced GR sensitivity in the periphery (16), aligning with findings from other laboratories as reviewed in (43). In the present study, we found that GR sensitivity was reduced by the antenatal treatment with GCs being lower in babies exposed out of phase. GR resistance has been associated with immune and mood disorders and although there is no evidence of a direct link, preterm children frequently suffer from both conditions (reviewed by (17, 44)).

Aiming to explore the molecular changes underlying the differential GR sensitivity, we first assessed the mRNA expression of *NR3C1 (GR)* and *FKBP5* in PBMCs, and we found no difference as described by others (16, 39). We reasoned that epigenetic changes such as methylation in key regulatory regions of both genes could still explain our observation (25, 38). Interestingly, we found significantly higher levels of methylation in an intronic regulatory region of the *FKBP5* gene, one of the main regulators of GR affinity to GCs and its translocation to the nuclei (45) in samples from babies exposed to GCs out of phase. This higher methylation does not significantly affect the expression of *FKBP5* itself, but it could affect the way in which GCs modulate downstream pathways (46).

We used predictive tools to identify the TFs likely binding to the hypermethylated CpGs of the *FKBP5* gene. We found that STAT1, 3, 4, and 5, NFATC, THAP1, and ZNF263 are binding in that region. Interestingly, these TFs are essential for the innate and adaptive immune response and for almost all of them, complex interactions with GR signaling pathways have been described (recently reviewed in (40)). For instance, STAT 1 and 3, NFATC, and THAP1 are indeed target genes of the GR (47). Reciprocally, cytokine signaling pathways including the signal transducer and activator of transcription (STATs), could regulate GR affinity and its translocation to the nuclei by influencing the expression of *FKBP5* (48, 49) as well as the DNA binding (50). This regulatory region of the *FKBP5* gene also contains binding sites for the nuclear factor of activated T cells, cytoplasmic, calcineurin-dependent (NFATC) and THAP domain containing, apoptosis-associated protein (THAP1). Proteins belonging to the NFATC family of TFs play a central role in inducible gene transcription during immune response, a major molecular target for immunosuppressive drugs (reviewed in (51)), while THAP1 has been linked to apoptosis induction (52).

The reciprocal interactions between GR and immune pathways are extremely complex: Every immune cell expresses the receptor, and it has been shown that the transcriptional output could lead to both immune suppression and immune activation depending on the cell type and context (11). Aiming to link the reduced GR sensitivity and the increased methylation in key regulatory regions of *FKBP5*, we assessed gene expression on a broader scale considering the circadian phase of GC exposure. Despite the intrinsic variability of the samples, we identified differential expression of genes involved in antibody production and B-cell function (eg, *IGHV, IGDH, CD19*), leukocyte migration and proliferation (eg, *MMP8, CXCL2, C5, SRC*), cytokine production (eg, *TLR10, CD40*), antigen presentation (eg, *HLA, LGMN, NPC2*), and cellular stress responses (eg, *PRDX1, SAMD, TRIM*), mainly downregulated in PBMCs from infants exposed to aGCs out of phase compared with those exposed in phase. Due to the nature of GC-GR signaling and the sample containing all blood cells, we did not observe changes in a single pathway or cell-type–specific effects. However, our data provide the basis to explore more specific targets in the future using high-throughput and single-cell–sequencing technologies. Lastly, we compared the frequency of lymphocytes between the groups and observed a lower frequency only of T_regs_ (CD4^+^CD25^+^FoxP3^+^) during the first 4 days of life of out-of-phase infants, which may represent reduced immune tolerance compared to the in-phase–exposed babies.

The main limitations of our study are the reduced number of samples analyzed and the intrinsic variability and reduced information about maternal conditions before and after birth. Although all samples were taken during the morning, the complexity of the study design and the availability of samples did not allow us to control for the number of days between the GC exposure and each infant’s birth. This aspect is especially relevant because the function of each immune cell is essentially circadian (53) and GCs are one of the main entrainment signals for the circadian clock. Thus, some of the effects we see could be caused by the timing of the exogenous GC exposure and induce mostly transient effects. Further studies should assess whether the immune function could be impaired in the long term, as we already observed at the behavioral level in 5-year-old children (16). Even if the effect were transient, our data suggest that the exposure to aGCs at the “wrong” time of day may reduce GR sensitivity and disrupt immune homeostasis and impair the capacity of the newborn to cope with immune challenges during the first days or weeks of life. Aligning GC administration with the maternal circadian rhythm could mitigate these effects and improve immune outcomes in a population of infants who are inherently prone to immune dysregulation and long-term immune impairment (44). Future clinical translation of circadian-based aGCs therapies will require strong evidence ensuring that the established short-term benefits, such as prevention of respiratory distress syndrome and associated morbidities are preserved, while the long-term risks are reduced. This becomes particularly relevant for the considerable proportion of infants who are exposed to aGCs but are delivered several weeks after the treatment or even at term, and are therefore exposed to increased risk with little or no therapeutic benefit. Our results provide a framework for evaluating this concept in larger, multicenter studies.

## Data Availability

All the scripts used in this study for data preprocessing and analysis are available on request. Raw data are deposited in Gene Expression Omnibus (GSE320139). All raw data included in the main figures and the supplemental information will be shared by the corresponding author on request.

## Abbreviations

aGCs: antenatal glucocorticoids
ANOVA: analysis of variance
bisDNA: bisulfite DNA
C: controls
DEX: dexamethasone
GCs: glucocorticoids
GO: gene ontology
GR: glucocorticoid receptor
GSEA: gene set enrichment analysis
IL-6: interleukin-6
IP: in-phase
IRoN: ImmunoRegulation of the Newborn
LPS: lipopolysaccharide
mRNA: messenger RNA
NFATC: nuclear factor of activated T cells, cytoplasmic, calcineurin-dependent
OP: out-of-phase
PBMCs: peripheral blood mononuclear cells
PI: premature infants
RT: room temperature
TF: transcriptions factor
THAP1: THAP domain containing, apoptosis-associated protein
T_regs_: regulatory T cells

## References

[1] Walani SR . Global burden of preterm birth. Int J Gynaecol Obstet. 2020;150(1):31‐33. 32524596 10.1002/ijgo.13195

[2] Lawn JE, Davidge R, Paul VK, et al Born too soon: care for the preterm baby. Reprod Health. 2013;10(S1):S5. 24625233 10.1186/1742-4755-10-S1-S5 PMC3828583

[3] Cheong JLY, Burnett AC, Treyvaud K, Spittle AJ. Early environment and long-term outcomes of preterm infants. J Neural Transm (Vienna). 2020;127(1):1‐8. 31863172 10.1007/s00702-019-02121-w

[4] Roberts D, Brown J, Medley N, Dalziel SR. Antenatal corticosteroids for accelerating fetal lung maturation for women at risk of preterm birth. Cochrane Database Syst Rev. 2017;3(3):CD004454. 28321847 10.1002/14651858.CD004454.pub3 PMC6464568

[5] Briceño-Pérez C, Reyna-Villasmil E, Vigil-De-Gracia P. Antenatal corticosteroid therapy: historical and scientific basis to improve preterm birth management. Eur J Obstet Gynecol Reprod Biol. 2019;234:32‐37. 30639954 10.1016/j.ejogrb.2018.12.025

[6] Alexander N, Rosenlöcher F, Stalder T, et al Impact of antenatal synthetic glucocorticoid exposure on endocrine stress reactivity in term-born children. J Clin Endocrinol Metab. 2012;97(10):3538‐3544. 22869608 10.1210/jc.2012-1970

[7] Ilg L, Kirschbaum C, Li SC, Rosenlöcher F, Miller R, Alexander N. Persistent effects of antenatal synthetic glucocorticoids on endocrine stress reactivity from childhood to adolescence. J Clin Endocrinol Metab. 2019;104(3):827‐834. 30285119 10.1210/jc.2018-01566

[8] Rakers F, Schleußner E, Muth I, et al Association between antenatal glucocorticoid exposure and the activity of the stress system, cognition, and behavior in 8- to 9-year-old children: a prospective observational study. Acta Obstet Gynecol Scand. 2022;101(9):996‐1006. 35652410 10.1111/aogs.14386 PMC9564447

[9] Räikkönen K, Gissler M, Kajantie E. Associations between maternal antenatal corticosteroid treatment and mental and behavioral disorders in children. JAMA. 2020;323(19):1924‐1933. 32427304 10.1001/jama.2020.3937 PMC7237984

[10] Solano ME, Holmes MC, Mittelstadt PR, Chapman KE, Tolosa E. Antenatal endogenous and exogenous glucocorticoids and their impact on immune ontogeny and long-term immunity. Semin Immunopathol. 2016;38(6):739‐763. 27465226 10.1007/s00281-016-0575-z

[11] Bhatt B, Franco LM. Endogenous glucocorticoids and human immunity: time to revisit old dogmas. Semin Immunol. 2025;78:101949. 40203674 10.1016/j.smim.2025.101949 PMC12146083

[12] Oster H, Challet E, Ott V, et al The functional and clinical significance of the 24-hour rhythm of circulating glucocorticoids. Endocr Rev. 2017;38(1):3‐45. 27749086 10.1210/er.2015-1080 PMC5563520

[13] Russell JA, Brunton PJ. Giving a good start to a new life via maternal brain allostatic adaptations in pregnancy. Front Neuroendocrinol. 2019;53:100739. 30802468 10.1016/j.yfrne.2019.02.003

[14] Moisiadis VG, Matthews SG. Glucocorticoids and fetal programming part 1: outcomes. Nat Rev Endocrinol. 2014;10(7):391‐402. 24863382 10.1038/nrendo.2014.73

[15] Astiz M, Oster H. Perinatal programming of circadian clock-stress crosstalk. Neural Plast. 2018;2018:5689165. 29593783 10.1155/2018/5689165 PMC5822916

[16] Astiz M, Heyde I, Fortmann MI, et al The circadian phase of antenatal glucocorticoid treatment affects the risk of behavioral disorders. Nat Commun. 2020;11(1):3593. 32681096 10.1038/s41467-020-17429-5 PMC7367845

[17] Lockett J, Inder WJ, Clifton VL. The glucocorticoid receptor: isoforms, functions, and contribution to glucocorticoid sensitivity. Endocr Rev. 2024;45(4):593‐624. 38551091 10.1210/endrev/bnae008 PMC11244253

[18] Lu NZ, Cidlowski JA. Translational regulatory mechanisms generate N-terminal glucocorticoid receptor isoforms with unique transcriptional target genes. Mol Cell. 2005;18(3):331‐342. 15866175 10.1016/j.molcel.2005.03.025

[19] Frank F, Liu X, Ortlund EA. Glucocorticoid receptor condensates link DNA-dependent receptor dimerization and transcriptional transactivation. Proc Natl Acad Sci U S A. 2021;118(30):e2024685118. 34285072 10.1073/pnas.2024685118 PMC8325269

[20] Akulov V, Jiménez Panizo A, Estébanez-Perpiñá E, van Noort J, Mashaghi A. Phosphorylation-regulated conformational diversity and topological dynamics of an intrinsically disordered nuclear receptor. J Phys Chem B. 2025;129(30):7719‐7730. 40673718 10.1021/acs.jpcb.5c03257 PMC12319912

[21] Baischew A, Engel S, Taubert MC, Geiger TM, Hausch F. Large-scale, in-cell photocrosslinking at single-residue resolution reveals the molecular basis for glucocorticoid receptor regulation by immunophilins. Nat Struct Mol Biol. 2023;30(12):1857‐1866. 37945739 10.1038/s41594-023-01098-1

[22] Moessmer P, Suren T, Majdic U, et al Active unfolding of the glucocorticoid receptor by the Hsp70/Hsp40 chaperone system in single-molecule mechanical experiments. Proc Natl Acad Sci U S A. 2022;119(15):e2119076119. 35377810 10.1073/pnas.2119076119 PMC9169861

[23] Paul SN, De Visser A, Motta F, et al Patterns of corticosterone exposure affect the subcellular localisation of mineralocorticoid and glucocorticoid receptor complexes and gene expression. Steroids. 2025;214:109524. 39490722 10.1016/j.steroids.2024.109524

[24] Boo SH, Shin MK, Ha H, Woo JS, Kim YK. Transcriptome-wide analysis for glucocorticoid receptor-mediated mRNA decay reveals various classes of target transcripts. Mol Cells. 2024;47(11):100130. 39426683 10.1016/j.mocell.2024.100130 PMC11577233

[25] Winkler BK, Lehnert H, Oster H, Kirchner H, Harbeck B. FKBP5 methylation as a possible marker for cortisol state and transient cortisol exposure in healthy human subjects. Epigenomics. 2017;9(10):1279‐1286. 28875708 10.2217/epi-2017-0057

[26] Klengel T, Mehta D, Anacker C, et al Allele-specific FKBP5 DNA demethylation mediates gene-childhood trauma interactions. Nat Neurosci. 2013;16(1):33‐41. 23201972 10.1038/nn.3275 PMC4136922

[27] Yehuda R, Daskalakis NP, Bierer LM, et al Holocaust exposure induced intergenerational effects on *FKBP5* methylation. Biol Psychiatry. 2016;80(5):372‐380. 26410355 10.1016/j.biopsych.2015.08.005

[28] Lee RS, Tamashiro KLK, Yang X, et al Chronic corticosterone exposure increases expression and decreases deoxyribonucleic acid methylation of Fkbp5 in mice. Endocrinology. 2010;151(9):4332‐4343. 20668026 10.1210/en.2010-0225 PMC2940504

[29] Mortazavi A, Williams BA, McCue K, Schaeffer L, Wold B. Mapping and quantifying mammalian transcriptomes by RNA-Seq. Nat Methods. 2008;5(7):621‐628. 18516045 10.1038/nmeth.1226 PMC13303166

[30] Fortmann I, Britsemmer JH, Lehmann M, et al Supplementary material for “The timing of antenatal glucocorticoids determines the receptor sensitivity in preterm infants”. Zenodo. 2026. 10.5281/zenodo.18656797 PMC13271807 41693150

[31] Austin PC, White IR, Lee DS, van Buuren S. Missing data in clinical research: a tutorial on multiple imputation. Can J Cardiol. 2021;37(9):1322‐1331. 33276049 10.1016/j.cjca.2020.11.010 PMC8499698

[32] Badia-i-Mompel P, Vélez Santiago J, Braunger J, et al Decoupler: ensemble of computational methods to infer biological activities from omics data. Bioinform Adv. 2022;2(1):vbac016. 36699385 10.1093/bioadv/vbac016 PMC9710656

[33] Müller-Dott S, Tsirvouli E, Vazquez M, et al Expanding the coverage of regulons from high-confidence prior knowledge for accurate estimation of transcription factor activities. Nucleic Acids Res. 2023;51(20):10934‐10949. 37843125 10.1093/nar/gkad841 PMC10639077

[34] Graspeuntner S, Lupatsii M, van Zandbergen V, et al Infants <90 days of age with late-onset sepsis display disturbances of the microbiome-immunity interplay. Infection. 2025;53(3):921‐934. 39541036 10.1007/s15010-024-02396-6 PMC12137456

[35] Pagel J, Hartz A, Figge J, et al Regulatory T cell frequencies are increased in preterm infants with clinical early-onset sepsis. Clin Exp Immunol. 2016;185(2):219‐227. 27163159 10.1111/cei.12810 PMC4955004

[36] Fortmann I, Dammann MT, Siller B, et al Infants younger than 90 days admitted for late-onset sepsis display a reduced abundance of regulatory T cells. Front Immunol. 2021;12:666447. 34512621 10.3389/fimmu.2021.666447 PMC8430331

[37] Gearing LJ, Cumming HE, Chapman R, et al CiiiDER: a tool for predicting and analysing transcription factor binding sites. PLoS One. 2019;14(9):e0215495. 31483836 10.1371/journal.pone.0215495 PMC6726224

[38] van der Knaap LJ, Oldehinkel AJ, Verhulst FC, van Oort FVA, Riese H. Glucocorticoid receptor gene methylation and HPA-axis regulation in adolescents. The TRAILS study. Psychoneuroendocrinology. 2015;58:46‐50. 25951242 10.1016/j.psyneuen.2015.04.012

[39] Merkulov VM, Merkulova TI, Bondar NP. Mechanisms of brain glucocorticoid resistance in stress-induced psychopathologies. Biochemistry (Mosc). 2017;82(3):351‐365. 28320277 10.1134/S0006297917030142

[40] Xu J, Wang B, Ao H. Corticosterone effects induced by stress and immunity and inflammation: mechanisms of communication. Front Endocrinol (Lausanne). 2025;16:1448750. 40182637 10.3389/fendo.2025.1448750 PMC11965140

[41] Jobe AH, Goldenberg RL, Kemp MW. Antenatal corticosteroids: an updated assessment of anticipated benefits and potential risks. Am J Obstet Gynecol. 2024;230(3):330‐339. 37734637 10.1016/j.ajog.2023.09.013

[42] Melville JM, Moss TJM. The immune consequences of preterm birth. Front Neurosci. 2013;7:79. 23734091 10.3389/fnins.2013.00079 PMC3659282

[43] Logan RW, McClung CA. Rhythms of life: circadian disruption and brain disorders across the lifespan. Nat Rev Neurosci. 2019;20(1):49‐65. 30459365 10.1038/s41583-018-0088-y PMC6338075

[44] Fortmann MI, Dirks J, Goedicke-Fritz S, et al Immunization of preterm infants: current evidence and future strategies to individualized approaches. Semin Immunopathol. 2022;44(6):767‐784. 35922638 10.1007/s00281-022-00957-1 PMC9362650

[45] Hartmann J, Bajaj T, Klengel C, et al Mineralocorticoid receptors dampen glucocorticoid receptor sensitivity to stress via regulation of FKBP5. Cell Rep. 2021;35(9):109185. 34077736 10.1016/j.celrep.2021.109185 PMC8244946

[46] Yin Y, Morgunova E, Jolma A, et al Impact of cytosine methylation on DNA binding specificities of human transcription factors. Science. 2017;356(6337):eaaj2239. 28473536 10.1126/science.aaj2239 PMC8009048

[47] ENCODE Project Consortium . The ENCODE (ENCyclopedia Of DNA Elements) project. Science. 2004;306(5696):636‐640. 15499007 10.1126/science.1105136

[48] Aittomäki S, Pesu M, Groner B, Jänne OA, Palvimo JJ, Silvennoinen O. Cooperation among Stat1, glucocorticoid receptor, and PU.1 in transcriptional activation of the high-affinity Fc gamma receptor I in monocytes. J Immunol. 2000;164(11):5689‐5697. 10820245 10.4049/jimmunol.164.11.5689

[49] Goleva E, Kisich KO, Leung DYM. A role for STAT5 in the pathogenesis of IL-2-induced glucocorticoid resistance. J Immunol. 2002;169(10):5934‐5940. 12421978 10.4049/jimmunol.169.10.5934

[50] Lerner L, Henriksen MA, Zhang X, Darnell JE. STAT3-dependent enhanceosome assembly and disassembly: synergy with GR for full transcriptional increase of the alpha 2-macroglobulin gene. Genes Dev. 2003;17(20):2564‐2577. 14522952 10.1101/gad.1135003 PMC218150

[51] Ulengin-Talkish I, Cyert MS. A cellular atlas of calcineurin signaling. Biochim Biophys Acta Mol Cell Res. 2023;1870(1):119366. 36191737 10.1016/j.bbamcr.2022.119366 PMC9948804

[52] Aguilo F, Zakirova Z, Nolan K, et al THAP1: role in mouse embryonic stem cell survival and differentiation. Stem Cell Reports. 2017;9(1):92‐107. 28579396 10.1016/j.stemcr.2017.04.032 PMC5511047

[53] Druzd D, Matveeva O, Ince L, et al Lymphocyte circadian clocks control lymph node trafficking and adaptive immune responses. Immunity. 2017;46(1):120‐132. 28087238 10.1016/j.immuni.2016.12.011 PMC5263259

